# 1-Methyl-1,2,3,4-Tetrahydroisoquinoline, an Endogenous Amine with Unexpected Mechanism of Action: New Vistas of Therapeutic Application

**DOI:** 10.1007/s12640-013-9402-7

**Published:** 2013-05-30

**Authors:** Lucyna Antkiewicz-Michaluk, Agnieszka Wąsik, Jerzy Michaluk

**Affiliations:** Department of Neurochemistry, Institute of Pharmacology Polish Academy of Sciences, Smetna Str.12, 31-343 Kraków, Poland

**Keywords:** TIQ, 1MeTIQ, Brain dopamine metabolism, Neuroprotection, Addiction, Rat

## Abstract

This review outlines the effects of 1,2,3,4-tetrahydroisoquinoline (TIQ) and its derivative, 1-methyl-1,2,3,4-tetrahydroisoquinoline (1MeTIQ), endogenous substances imbued with high pharmacological potential and broad spectrum of action in brain. 1MeTIQ has gained special interest as a neuroprotectant, and its ability to antagonize the behavioral syndrome produced by well-known neurotoxins (e.g., MPTP; rotenone). This review is thus focused on mechanisms of action of 1MeTIQ in behavioral, neurochemical, and molecular studies in rodents; also, effects of TIQ and 1MeTIQ on dopamine metabolism; and neuroprotective properties of TIQ and 1MeTIQ in vitro and in vivo. Finally, antiaddictive properties of 1MeTIQ will be described in cocaine self-administered rats. Findings implicate TIQ and especially its methyl derivative 1MeTIQ in unique and complex mechanisms of neuroprotection in various neurodegenerative illnesses of the central nervous system. We believe that MAO inhibition, free radicals scavenging properties, and antagonism to the glutamatergic system may play an essential role in neuroprotection. In addition, the results strongly support the view that 1MeTIQ has a considerable potential as a drug for combating substance abuse, through the attenuation of craving.

## Introduction

1,2,3,4-Tetrahydroisoquinoline (TIQ) is a member of a family of tetrahydroisoquinolines widespread in plant and animal and human brains (McNaught et al. 1998; Rommelspacher and Susilo 1985). In most cases, tetrahydroisoquinolines can be formed as condensation products of biogenic amines (i.e., phenylethylamines and catecholamines) with aldehydes or α-keto acids by the so-called Pictet–Spengler reaction (Rommelspacher and Susilo 1985; Zarranz de Ysern and Ordonez 1981; Nagatsu 1997; McNaught et al. 1998), although some of them may be also synthesized enzymatically (Yamakawa and Ohta 1997, 1999; Naoi et al. 2004). The tetrahydroisoquinoline family can be divided into compounds with catechol- and non-catechol structure. TIQ is the simplest representative of the group of non-catechol tetrahydroisoquinolines which occur naturally in plants and in a variety of food products (Makino et al. 1988; Niwa et al. 1989) as well as in the brain of humans, primates, and rodents (Kohno et al. 1986; Makino et al. 1988; Niwa et al. 1987; Ohta et al. 1987; Yamakawa et al. 1999).

For the first time, tetrahydroisoquinolines attracted considerable attention of neurochemists and pharmacologists when Davis and Walsh (1970) demonstrated that the alcohol metabolite acetaldehyde promoted in vitro conversion of [^14^C]dopamine into [^14^C]tetrahydropapaveroline (THP). Simultaneously, THP was identified in the urine of parkinsonian patients on l-DOPA (3,4-dihydroxyphenylalanine) medication (Sourkes 1971; Sandler et al. 1973; Matsubara et al. 1992) and in the urine and brain of rats treated with l-DOPA (Turner et al. 1974). Almost at the same time, salsolinol (6,7-dihydroxy-1-methyl-1,2,3,4-TIQ), an adduct of dopamine and acetaldehyde, was identified in the urine of non-pathologic human volunteers, occurring at high concentrations in the urine of intoxicated alcoholics (Collins et al. 1979) and in brains of rats treated with ethanol (Collins and Bigdeli 1975). Although TIQ has been proposed to be one of the etiological factors of Parkinson’s disease (PD), its implication in the pathogenesis is not clear, in contrast to other tetrahydroisoquinolines with rather neurotoxic mechanism of action in the brain, e.g., salsolinol or 1-benzyl-1,2,3,4-TIQ (1BnTIQ).

Early studies on tetrahydroisoquinolines revealed their neuroleptic-like properties (Ginos and Doroski 1979) and our more recent results suggest that TIQ and its derivatives are antagonists of agonistic conformation of the dopamine D_2_ receptor (Antkiewicz-Michaluk et al. 2007; Vetulani et al. 2001, 2003a). This explains why TIQ and its congeners effectively block dopaminergic stimulation without affecting much the basal locomotor activity. Pharmacologically tetrahydroisoquinolines aroused also an interest as potential NMDA (*N*-methyl-d-aspartate) receptor antagonists (Ortwine et al. 1992). Some of them were described as effective antagonists of the phencyclidine (PCP) site (Rogawski et al. 1989). However, most tetrahydroisoquinolines do not substitute for PCP (Nicholson and Balster 2003).

Apart from TIQ, this group encompasses also, the methyl derivative of TIQ, 1-methyl-1,2,3,4-TIQ (1MeTIQ), is a neuroprotective compound. Among several endogenous TIQs 1MeTIQ has a special position, as very early it was described in the brain (Kohno et al. 1986; Makino et al. 1990; Niwa et al. 1987; Ohta et al. 1987), and shortly thereafter recognized as a potential antiparkinsonian agent on the basis of reversal of bradykinesia induced by 1-methyl-4-phenyl-1,2,3,6-tetrahydropyridine (MPTP), TIQ, or 1BnTIQ (Tasaki et al. 1991; Kotake et al. 1995). 1-MeTIQ was identified in normal rat brains in 1986 (Kohno et al. 1986), and subsequently found to be present in foods rich in 2-phenylethylamine, from which it may enter the brain (Makino et al. 1988). However, it is also synthetized enzymatically in the brain from 2-phenethylamine to pyruvate (Niwa et al. 1990; Tasaki et al. 1993; Yamakawa and Ohta 1997). Having an asymmetric carbon atom, 1MeTIQ may appear in the form of R- and S-stereoisomers, and the product found in brain and in foods is a racemate (Makino et al. 1990), although the stereoisomers do not much differ in their biological actions (Abe et al. 2001; Wąsik et al. 2012). 1MeTIQ unlike several other TIQs displays neuroprotective and antiaddictive properties which will be particularly emphasized in this article (Antkiewicz-Michaluk and Vetulani 2001; Antkiewicz-Michaluk et al. 2003, 2004, 2006; Wąsik et al. 2007, 2010).

This article reviews some important aspects concerning the chemistry, distribution, pharmacology, and mechanism of action of TIQ and its methyl derivative, 1MeTIQ the simplest representatives of the unsubstituted non-catechol tetrahydroisoquinolines in the mammalian brain.

## Synthesis of 1MeTIQ in the Brain

1MeTIQ was identified in normal rat brains by Kohno and coworkers (Kohno et al. 1986), and subsequently found to be present in foods rich in 2-phenylethylamine, which readily penetrates into the brain across the blood–brain barrier (Makino et al. 1988). 1MeTIQ may be also synthesized enzymatically in the brain (Niwa et al. 1990). The enzyme involved in that process called 1MeTIQase was localized in the mitochondrial-synaptosomal fraction of rat brain, isolated, and purified (Niwa et al. 1990; Tasaki et al. 1993). Its activity is spread throughout the brain, the highest activity being observed in the dopaminergic areas that are implicated in the etiology of PD [striatum and substantia nigra (SN)] and in the cortex. During aging the activity of 1MeTIQase falls (by approximately 40–50 %) in the areas of its highest activity (Absi et al. 2002). 1MeTIQase may be important in the pathogenesis of PD. The cerebral concentration of 1MeTIQ in normal rat brains was recently determined as 3.5 ng/g tissue, exceeding several times (three- to fivefold) the concentrations of other simple tetrahydroisoquinolines (Inoue et al. 2008). Most of the studies on 1MeTIQ were carried out on the brains of rodents, but the results on monkeys demonstrate that the regional distribution of 1MeTIQ, other simple tetrahydroisoquinolines, and 1MeTIQase activity are correlated (Yamakawa et al. 1999).

It was also demonstrated that 1MeTIQ synthesis is inhibited by agents that induce experimental parkinsonism (Igarashi et al. 1999; Tasaki et al. 1991; Yamakawa and Ohta 1999). Interestingly, the 1MeTIQ concentration in SN declines in parkinsonian patients as well as in aged rats, by as much as 50 % (Ayala et al. 1994). All those data indicate that the change in 1MeTIQ content of brain may play an important role in the pathogenesis of the toxin-induced parkinsonism, and that the degeneration of dopaminergic neurons may proceed as a result of the loss of neuroprotection afforded by 1MeTIQ. Thus, it seems that 1MeTIQ is an endogenous substance that protects mainly dopamine cells against free radical damage.

## Neuroprotection

### Free Radical Scavenging Properties Afforded by TIQ and 1MeTIQ

To determine whether TIQ and 1MeTIQ may protect against oxidative stress we investigated their capacity to inhibit hydroxyl radical generation in vitro. Oxidative stress leads to the production of reactive oxygen species (Harman 1981), such as superoxide anion radical and hydroxyl radical (^·^OH)—chemical species known to damage cellular macromolecules (e.g., lipids, sugars, proteins), and this damage can lead to equally damaging secondary products (Sayre et al. 2008). Based on these data, oxidative stress was long regarded as a universal mechanism of inducing cell death (Dykens 1999). In the brain, the main source of toxic ^·^OH formation and H_2_O_2_ generation is MAO, consequent to monoamine deamination (for review see Singer and Ramsay 1995). Similarly, excessive intraneuronal dopamine catabolism by MAO augments the formation of free radicals in the brain. A direct study on the free radical scavenging capacity of other tetrahydroisoquinolines was carried out in vitro in rodent brain. Both TIQ and 1MeTIQ, in contrast to other tetrahydroisoquinolines (e.g., 1BnTIQ and salsolinol), inhibit MAOA and MAOB activities and possess other antioxidant properties (Patsenka and Antkiewicz-Michaluk 2004), as indicated by the effects of TIQ and 1MeTIQ to inhibit free radical formation and abolish dopamine generation of ^·^OH via the Fenton reaction (Antkiewicz-Michaluk et al. 2006). Those results demonstrate that TIQ and 1MeTIQ, independent of direct interaction with biological structures, possess intrinsic antioxidant properties.

### Neuroprotection in Relation to Dopaminergic Mechanisms in In Vitro and Ex Vivo Studies

Interaction with presynaptic dopamine receptors was investigated by studying 1MeTIQ-induced displacement of dopamine receptor ligands from their binding sites. In general, tetrahydroisoquinolines do not displace antagonistic ligands bound to dopamine D_2_ receptors (Antkiewicz-Michaluk et al. 2007; Vetulani et al. 2003a), the exception being [^11^C]raclopride. Depression of binding of [^11^C]raclopride may be interpreted as the sign of increased synaptic dopamine concentrations, which competes with raclopride at dopamine D_2_ receptor sites (Laruelle 2000). Tetrahydroisoquinolines were shown to displace [^11^C]raclopride, and the (S)-enantiomers of TIQ and 1MeTIQ were most potent in this respect. These findings suggest that tetrahydroisoquinoline analogs profoundly stimulate dopamine release, resulting in the competitive inhibition of [^11^C]raclopride binding to dopamine D_2_ receptors, but not loss of receptor number (Ishiwata et al. 2001). The dopamine receptor agonist [^3^H]apomorphine was another ligand displaced from dopamine D_2_ receptors by tetrahydroisoquinolines (Antkiewicz-Michaluk et al. 2007; Vetulani et al. 2003a). In contrast to antagonists, an agonist radioligand binds preferentially to the high-affinity state and is expected to have greater sensitivity to dopamine, the endogenous agonist. Thus, the experiments with [^3^H]apomorphine displacement confirm that tetrahydroisoquinolines may release dopamine from dopaminergic terminals. However, owing to their MAO-inhibiting properties tetrahydroisoquinolines do not cause neurodegeneration of dopaminergic neurons.

In biochemical studies, MPTP and pro-parkinsonian β-carbolines potently inhibited the activity of 1MeTIQ-ase (Yamakawa and Ohta 1999). It is well established by behavioral, biochemical ex vivo but also in vivo microdialysis studies, that both enantiomers (R)- and (S)- as well as racemic (R,S)-1MeTIQ demonstrate neuroprotective activity, as evidenced by their attenuation of the behavioral and biochemical effects of dopaminergic neurodegeneration induced by experimental neurotoxins such as: MPTP, 1BnTIQ, and rotenone (Antkiewicz-Michaluk et al. 2003, 2004, 2011; Kotake et al. 1995, 2005; Tasaki et al. 1991).

Several tetrahydroisoquinolines and their congeners, including TIQ and 1MeTIQ, interfere with MAO activity, indicating putative neuroprotection relating to the pathogenesis of PD (Naoi and Maruyama 1993). Subsequently, Thull et al. (1995) investigated 45 isoquinoline derivatives and found most of them to be reversible inhibitors of MAOA and MAOB, with preferential effects on the A form. Their studies brought to the forefront the question of the physiological significance of endogenous MAO inhibitors, and a suggested role for endogenous tetrahydroisoquinolines in the control of neurotransmitter function, and prevention of neurotoxicity related to MAO activity in the brain.

The data from ex vivo neurochemical experiments have shown stereospecificity of 1MeTIQ enantiomers, (R)- and (S)- in respect of their effects on dopamine catabolism. While both enantiomers increased the concentrations of dopamine and its extraneuronal metabolite, 3-methoxytyramine (3-MT) in rat striatum, they differently affected dopamine catabolism. Thus, (R)-1MeTIQ increased both the level of the final dopamine metabolite homovanillic acid (HVA) (by about 70 %) and the rate of dopamine metabolism (by 50 %), while (S)-1MeTIQ depressed the DOPAC (3,4-dihydroxyphenylacetic acid) and HVA levels (by 60 and 40 %, respectively), and attenuated the rate of dopamine metabolism (Antkiewicz-Michaluk et al. 2011). These data suggest that the (S)-enantiomer may offer better and more effective protection against neurotoxicity. It would be important to mention that even after chronic administration a high dose of 1MeTIQ never produced noxious effects on dopamine neurons (Antkiewicz-Michaluk et al. 2001).

Showing structural resemblance to MPTP, the potent neurotoxin capable of producing persistent parkinsonism in humans (Langston et al. 1983) and in laboratory animals (Jenner and Marsden 1986), initially all tetrahydroisoquinolines were assumed to be neurotoxic to dopamine neurons. In fact, the early studies reported that they generally are neurodegenerating agents (Suzuki et al. 1990), the most neurotoxic being 1BnTIQ, and *N*-methyl derivatives, (R)-1,2-dimethyl-5,6-dihydroxy-TIQ, (R)-*N*-methyl-salsolinol, and TIQ (Nagatsu 1997). This finding contrasted with an earlier report which found no neurotoxicity of tetrahydroisoquinolines on nigrostriatal dopamine neurons (Perry et al. 1988). The most recent studies, in which the actions of 1MeTIQ and TIQ were directly compared, suggest that TIQ, in fact, produces some damage to dopaminergic neurons, as reflected by a mild but significant decrease in the striatal dopamine concentration in rats chronically administered TIQ in high doses (50–100 mg/kg). In contrast, 1MeTIQ has never been shown to produce a decline in dopamine in brain, although both of these tetrahydroisoquinolines similarly affect dopamine catabolism (Antkiewicz-Michaluk et al. 2000a; 2001; Antkiewicz-Michaluk and Vetulani 2001).

### Neuroprotection of 1MeTIQ Against Rotenone

Rotenone, a natural compound, is a classical, lipophilic inhibitor of mitochondrial complex I (Gutman et al. 1970; Horgan et al. 1968), and selectively toxic to dopaminergic neurons (Marey-Semper et al. 1993). Rotenone, an environmental toxin induces the formation of Lewy bodies, which are the most characteristic histopathological feature of Parkinson’s disease (Betarbet et al. 2000), and may be used to produce a parkinsonian syndrome more realistic from MPP^+^ animal modeling of PD. A defect of mitochondrial function due to complex I inhibition was postulated to be the cause of rotenone-induced neurodegeneration (Jenner 2001; Greenamyre et al. 2001). Rotenone also causes dopamine release, as evidenced by microdialysis and neurochemical data (Santiago et al. 1995; Thiffault et al. 2000), and this may also contribute to the degeneration of dopaminergic neurons. In our studies, rotenone administered in a single dose did not produce evident behavioral or biochemical effect. In contrast, repeated administration of rotenone (12 mg/kg s.c.) causing abnormalities in general behavior produced considerable mortality and dramatic increases in dopamine metabolism, which may be ascribed to an increase in the oxidative pathway, and strongly depressed the concentration of the extracellular dopamine metabolite, 3-MT. These behavioral and biochemical changes were effectively counteracted by administration of 1MeTIQ before each dose of rotenone (Antkiewicz-Michaluk et al. 2003). In addition, rotenone administered intracerebrally to the left medial forebrain bundle (MFB) produced neurodegeneration of dopamine neurons in extrapyramidal system (a considerable decrease in dopamine and its metabolite levels) without affecting the serotonin system (Antkiewicz-Michaluk et al. 2004). Those changes were observed 21 days after the intracerebral injection of rotenone, they suggest a durable neurotoxic effect. Peripheral administration of 1MeTIQ (50 mg/kg i.p.) before, and then daily for 21 days, significantly reduced the fall of striatal dopamine concentration (Antkiewicz-Michaluk et al. 2004). The above data suggest that 1MeTIQ is able to counteract the damaging action of dopaminergic neurotoxin, rotenone and seems to be a potential neuroprotective agent.

### Neuroprotection of 1MeTIQ Against Glutamate-Evoked Neurotoxicity

Recently, it was demonstrated that 1MeTIQ shares many activities with TIQ, and found that the compounds similarly inhibit free radical generation in an abiotic system, as well as indices of neurotoxicity (caspase-3 activity and lactate dehydrogenase release) induced by glutamate in mouse embryonic primary cell cultures (Antkiewicz-Michaluk et al. 2006). However, in granular cell cultures obtained from 7-day-old rats, 1MeTIQ (in concentration-related manner) prevented glutamate-induced cell death and ^45^Ca^2+^ influx, whereas TIQ did not. Such profile of action of 1MeTIQ suggested specific effects of this compound on an excitatory amino acids receptor. In addition, it was shown in an in vivo microdialysis experiment that 1MeTIQ prevents kainate-induced release of excitatory amino acids from the rat frontal cortex (Antkiewicz-Michaluk et al. 2006).

Comparing the chemical structure of 1MeTIQ with other known compounds containing TIQ skeleton and their molecular mechanism of action, one can find similarities between 1MeTIQ and these derivatives which are non-competitive AMPA/kainate receptor antagonists that protect animals in the maximal electroshock seizure, pentylenetetrazole and audiogenic DBA/2 mouse seizure models (Ferreri et al. 2004; Gitto et al. 2003). In fact, 1MeTIQ exerts anticonvulsant effects, increasing the threshold for electroconvulsions and potentiation of the antiseizure action of carbamazepine and valproate against maximal electroshock (Luszczki et al. 2006). In the light of all these experiments, 1MeTIQ offers a unique and complex mechanism of neuroprotection in which inhibitory effect on MAO connected with free radicals scavenging properties, and antagonism to the glutamatergic system seems to play a very important role.

## Addiction

Addiction is a complex disease process of the brain which results from recurring drug intoxication and is modulated by genetic, experiential, and environmental factors. Drug addiction is one of the most difficult medical and social problems, as no effective pharmacotherapy has been available so far. Until recently, it was believed that addiction was associated with neuroplasticity in the cortico-striatal brain circuitry, which is important for adaptive behavior and predominantly involved reward processes mediated by limbic circuits, whereas results from recent neuroimaging studies have implicated additional brain areas, especially the frontal cortex (Goldstein and Volkov 2002). Drug addiction is often defined by the pharmacological terms: *tolerance*, *sensitization*, *dependence, and withdrawal.* *Tolerance* refers to the phenomenon where repeated administration of a drug at the same dose causes a diminishing effect or a need for an increasing drug dose to produce the same effect. *Sensitization* refers to the opposite condition where repeated administration of the same drug dose produces an escalating effect. Interestingly, the same drug can simultaneously evoke tolerance and sensitization to its numerous diverse effects (e.g., in the case of morphine, tolerance to its analgesic effect and sensitization to its locomotor effect). *Dependence* is defined as a need for continual drug exposure to avoid a *withdrawal* syndrome which is characterized by physical or motivational disturbance when the drug is withdrawn.

The neurobiological changes that accompany drug addiction have not been understood so far; however, drugs of abuse are unique in terms of their reinforcing properties. Dopaminergic mechanisms are a traditional target in the field of addiction, as the acute rewarding effects of addictive drugs are mediated by enhancing dopamine transmission; moreover, dopamine release reinforces reward learning (Berridge and Robinson 1998; Kelley 2004a, b). A question arises about the neurobiological substrate of reward. The nucleus accumbens (NAc) as a ventral striatum is considered to be a crucial point of integration of information by receiving emotional and cognitive inputs, and by projecting to motor output regions (Mogenson et al. 1980; Kelley 2004a). The NAc, along with the hippocampus, frontal cortex (FCx), and basolateral amygdala, receives dopamine input from the ventral tegmental area (VTA); furthermore, as it has been shown by many others, the majority of dopamine neurons that innervate the forebrain are located in the midbrain, specifically in the VTA and SN (Fallon and Loughlin 1995; Pitkanen 2000). The SNc innervates the dorsal striatum (caudate–putamen), whereas the VTA provides an input to the rest of the forebrain, including the ventral striatum (NAc), FCx, amygdala, and hippocampus. Early theories on drugs of abuse and natural rewards suggested that activation of dopamine neurons in VTA, and the release of dopamine in target structures signaled reward, especially in the NAc (Di Chiara 2002; Ungless 2004). However, aversive stimuli also increase dopamine release in a variety of brain structures, which indicates a role of dopamine beyond reward (Inglis and Moghaddam 1999). It is noteworthy that some evidence points to differential dopamine responses to aversive versus rewarding stimuli (Schultz 2002, 2010). Some recent studies have also shown that the glutamate system and its release is an important factor in drug addiction, and that imbalance in glutamate homeostasis engenders changes in neuroplasticity, which impair communication between the prefrontal cortex and the NAc (Kalivas 1995; Ma et al. 2006; Nagy 2004; Popik et al. 1998). In a clinical setting, neuroimaging studies have shown that cue or drug exposure increased the activity of FCx and NAc, as well as self-reported drug craving in cocaine addicts (Goldstein and Volkov 2002). In animal models, a challenge of cocaine or heroin increases the synaptic release of glutamate in cocaine- or heroin-withdrawn rats as a result of the activation of corticostriatal pathways; and on the other hand, inactivation of the corticostriatal pathway has been shown to be effective in inhibiting cocaine- or heroin-induced drug seeking behavior (Kalivas et al. 2005).

### Cocaine Addiction: The Effect of 1MeTIQ

Regardless of the mechanism of action of drugs of abuse, the essential role of the mesolimbic dopaminergic system in addiction has been well established (Goldstein and Volkov 2002; Grimm et al. 2003; Moore et al. 1998a, b); to this end, several antidopaminergic drugs were tested as potential anti-abuse agents (Berger et al. 1996; Smelson et al. 2004). Although neuroleptics were previously found not to be useful in this respect, partial agonists of the dopamine D_2_ and D_3_ receptor aroused some hopes (Campiani et al. 2003; Le Foll et al. 2005; Mach et al. 2004). Furthermore, a dopamine reuptake inhibitor could be expected to partially substitute for cocaine and other drugs of abuse, hence self-administration would be diminished and craving minimized (Ritz et al. 1987; Wilcox et al. 2000; Carroll et al. 2004). This type of substitution pharmacotherapy has been found to be highly effective in the treatment of nicotine and heroin addiction (methadone). Hence, studies of partial agonists with an antidopaminergic profile of action different from that of classic neuroleptics seem justified. In the light of the above data, 1MeTIQ is an interesting candidate for future clinical studies.

A vast body of evidence indicates that, apart from the dopaminergic system, also the glutaminergic system is involved in the addiction to drugs of abuse. Hence, of special interest are the observations that 1MeTIQ antagonizes the kainate-induced release of glutamate and aspartate in rat FCx and shows neuroprotection against the excitotoxicity produced by glutamate in cultured cells (Antkiewicz-Michaluk et al. 2006). In addition, 1MeTIQ antagonizes the MK-801-produced behavioral and neurochemical effects (Pietraszek et al. 2009) and shares tolerance to excitotoxicity in rat with some well-established uncompetitive NMDA receptor antagonists (Kuszczyk et al. 2010). The latest results reveal a new mechanism of the 1MeTIQ-evoked neuroprotection based on the induction of neuronal tolerance to excitotoxicity.

It is well known that acute administration of the drugs of abuse: cocaine, amphetamine (psychostimulants), and opiates increases the locomotor activity of animals. Repeated administration of the drug of abuse induces neurobiological changes, such that after 10 days of withdrawal, acute administration of the drug produces an even greater increase in locomotor activity, a process called sensitization. Both behavioral sensitization, self-administration, and drug-reinstatement of seeking behavior are the major models of drug addiction (Pierce and Kalivas 1997). The compounds which antagonize locomotor sensitization and self-administration in animals may demonstrate anti-addictive properties in a clinic (Narayanan et al. 1996).

Exogenously applied 1MeTIQ significantly antagonized cocaine-induced locomotor sensitization, cocaine self-administration, and cocaine-induced reinstatement of seeking behavior (Filip et al. 2007; Wąsik et al. 2010). The phenomenon is of interest, as it might be caused by the same mechanisms as those responsible for psychoses or craving for drugs of abuse in humans abusing cocaine or other psychostimulants (Robinson and Berridge 1993; Segal et al. 1981). Both clinical and preclinical studies indicate that the behavioral response to cocaine, including the discriminative stimulus and rewarding effects as well as reinstatement of cocaine seeking behavior, depends on the drug’s ability to block the dopamine transporter (Di Chiara 1995; Heidbreder and Hagan 2005). As 1MeTIQ produced parallel decreases in cocaine self-administration and cocaine-induced relapse, the compound may suppress the motivation for drug seeking by decreasing the reinforcing effects of cocaine, and generally by attenuation of the reinforcing effect of drugs of abuse. In fact, activation of both the dopaminergic and the glutaminergic systems has significance in altering the maintenance of cocaine self-administration (Cornish et al. 1999; Pulvirenti et al. 1992), and drug-priming-induced reinstatement of cocaine seeking (Ito et al. 2002; Kalivas and McFarland 2003).

1MeTIQ’s inhibitory mechanism on cocaine maintained responding and relapse may include complex interactions with both dopaminergic and/or glutaminergic transmission.

### Neurochemical Changes Produced by 1MeTIQ in Cocaine-Dependent Rats

Cocaine, a potent inhibitor of monoamine transporters, belongs to the most powerful addictive substances in humans and its abuse has a high risk of relapse (Gawin 1991). Studies on the involvement of biogenic amines in cocaine addiction have shown a contribution of dopamine and serotonin to the maintenance of cocaine self-administration, extinction, and reinstatement of drug-seeking behavior. Dopamine was the amine investigated the most extensively, possibly because of its assumed role as the main neurotransmitter of reward (Di Chiara et al. 2004). It was demonstrated by several authors during cocaine self-administration, that extracellular concentrations of dopamine and serotonin in the striatum increase (Di Ciano et al. 1995; Gratton and Wise 1994; Wąsik et al. 2010), but the rate of monoamines metabolism and the concentrations their metabolites in brain structures decrease (Antkiewicz-Michaluk et al. 2006). The functional significance of the observed depression of the level of monoamine metabolites requires explanation. The determination of changes in metabolism rate yields information about efficiency of the neurotransmitter system. Depending on the state of receptor and the rate of synthesis of a neurotransmitter, the changes in metabolite levels in the same direction may have different consequences. Activation of a receptor (e.g., dopamine receptor) during stabilized self-administration of cocaine would result in a feedback inhibition of neurotransmitter release by activation of autoreceptors, and the depression of neurotransmitter metabolites without significant changes in neurotransmitter concentration in the neuron. This is reflected by a decrease in the neurotransmitter metabolism index. As the stimulation is indirect, through inhibition of neurotransmitter reuptake, the interaction between the neurotransmitter and receptor is enhanced rather than depressed. On the contrary, during cocaine withdrawal, dopamine and noradrenaline concentrations as well as concentration of their metabolites were diminished, suggesting cocaine-induced impairment in the function of catecholamine neurons, and a consequent decrease in the synthesis and release of neurotransmitters (Parsons et al. 1995; Weiss et al. 1992; Antkiewicz-Michaluk et al. 2006).

While catecholamines seem to be involved in cocaine addiction, the role of serotonin seems to be limited. Specific serotonin agonists do not seem to have significant reinforcing efficacy (Locke et al. 1996). Clinical findings also indicate inefficiency of serotonergic manipulation in combating cocaine dependence (Lima et al. 2003). Although, serotonin metabolism is inhibited in the presence of cocaine, in contrast to alterations in catecholaminergic system, the change was transient.

Basically, a chronic cocaine self-administration similarly to passive administration suppresses the metabolism—both synthesis and release of monoamines in several brain structures (Karoum et al. 1990; Trulson and Ulissey 1987; Antkiewicz-Michaluk et al. 2006). The changes in catecholamine metabolism persist for a considerable period after cessation of cocaine self-administration, suggesting a long-lasting functional impairment of dopamine and noradrenaline systems. In contrast, changes in the serotonergic system are transient, showing the lack of involvement of serotonin in long-term consequences of cocaine exposure (Antkiewicz-Michaluk et al. 2006).

It is suggested that 1MeTIQ is a potential anti-abuse agent, as the drugs which reduce cocaine seeking behavior also reduce cocaine craving (Fuchs et al. 1998; Baker et al. 2001). The possible anti-abuse properties of 1MeTIQ are particularly interesting, as the compounds of this group are proposed to act as regulators of brain homeostasis (Antkiewicz-Michaluk et al. 2000a, b; Vetulani 2001). The question arises whether 1MeTIQ can reach the brain in concentrations producing pharmacological effects? In contrast to catechol TIQs (e.g., salsolinol), non-catechol TIQs such as TIQ and 1MeTIQ penetrate to the brain easily and induce a variety of central effects. No direct measurements of 1MeTIQ concentration in the brain after peripheral administration of the compound have been carried out, but its close congener, TIQ after administration of 40 mg/kg reached the concentration of 250 nmole/g in rat (Lorenc-Koci et al. 2004).

In cocaine-dependent rats receiving a priming dose of cocaine in the presence of previously administered 1MeTIQ, the concentration of dopamine in limbic structures was significantly higher than in rats receiving cocaine alone. It might be assumed that the blockade of reinstatement by 1MeTIQ is related to this effect (Antkiewicz-Michaluk et al. 2007). There is the long established view that depression of dopaminergic activity in the limbic structures may be responsible for craving (Rossetti et al. 1992; Little et al. 1996). Because 1MeTIQ elevates the concentration of dopamine preferentially in limbic structures (NAc) in cocaine-dependent rats, and at the same time inhibits dopamine metabolism in structures containing cell bodies (SN, VTA), 1MeTIQ may be responsible for its inhibition of reinstatement (Antkiewicz-Michaluk et al. 2007).

Another neurochemical action of 1MeTIQ, possibly related to its anticraving effect, is activation of the noradrenergic system in the brain. This effect may be related to the antagonistic action of 1MeTIQ on alpha-2-adrenoceptors. Such an activity was described previously for other, closely related TIQs (Michaluk et al. 2002; Vetulani et al. 2003a, b). The ability of 1MeTIQ to increase the level of the main metabolite of noradrenaline in CNS, 3-methoxy-4-hydroxyphenylglycol (MHPG), as well as its extraneuronal metabolite normetanephrine (NM), reflects the antagonistic effect of 1MeTIQ on the alpha-2-adrenoceptor (Antkiewicz-Michaluk, unpublished data). Inhibition of alpha-2-adrenoceptors would result in an increase in noradrenaline release from the nerve endings, and consequently activation of the noradrenergic system.

In the light of the recent experimental data it looks as though serotonin plays a less important role in the phenomenon of cocaine reinstatement. It was shown that cocaine depresses serotonin metabolism only transiently, and that the changes do not persist throughout the withdrawal period, in contrast to dopamine and noradrenaline systems (Antkiewicz-Michaluk et al. 2007).

### Morphine Addiction and the Effect of 1MeTIQ

Morphine acts through activation of opioid μ-receptors and produces the antinociceptive effect called analgesia. It is well known that activation of opioid μ-receptors is closely related with inhibition of calcium uptake and this process is responsible for opioid-induced analgesia (Kamikubo et al. 1983; Chapman and Way 1982). 1MeTIQ administered alone was shown to have a slight antinociceptive effect in the “hot-plate” test in rats, but co-administration of morphine strongly potentiated its analgesia (Wąsik et al. 2007; Vetulani et al. 2003a, b). Moreover, 1MeTIQ applied before each morphine injection completely inhibited the development of morphine tolerance, and prevented naloxone-induced precipitation of the abstinence syndrome (head-twiches and body weight loss) in morphine-dependent rats (Wąsik et al. 2007). A question arises as to the mechanism responsible for 1MeTIQ-induced potentiation of morphine-analgesia, prevention of morphine-produced tolerance, and abstinence syndrome. Some have postulated that the activity of MAO, the enzyme crucial for monoamine and special dopamine catabolism, and the production of free radicals, play an important role in opiate analgesia, tolerance, and dependence (Garzon et al. 1979; Grassing and He 2005). In fact, the irreversible MAOB inhibitor deprenyl and other antioxidants such as vitamin C produced an increase in morphine antinociception and prevention of the development of morphine tolerance and physical dependence in rodents (Sánchez-Blázquez et al. 2000; Khanna and Sharma 1983). 1MeTIQ, a neuroprotective substance, inhibits MAO and possesses free radical scavenging properties (Antkiewicz-Michaluk et al. 2006). This mechanism would be partially responsible for its antinociception and antiaddictive effects. In addition, it has been shown that 1MeTIQ is also effective in prevention of morphine-induced place preference and alcohol intake (Antkiewicz-Michaluk et al. 2005).

Moreover, others have shown that morphine did not effectively trigger the processes leading to development of tolerance and dependence when administered during Ca^2+^ channel blockade. Blockade of the voltage-dependent l-type Ca^2+^ channels effectively facilitates the analgesic action of morphine and prevents the behavioral and neurochemical signs of naloxone-precipitated abstinence syndrome (Contreras et al. 1988; Del Pozo et al. 1987; Michaluk et al. 1998). NMDA glutamate receptor activation and associated Ca^2+^ influx may also be involved in the induction of morphine sensitization (Vanderschuren and Kalivas 2000). It should be taken into account that 1MeTIQ prevented glutamate-induced cell death and ^45^Ca^2+^ influx in granular cell cultures (Antkiewicz-Michaluk et al. 2006). Thus, 1MeTIQ besides the inhibitory influence on the activity of MAO and free radical scavenging properties possesses mild activity at NMDA receptors.

## Conclusions

The experimental data assembled in this review allow for a more precise characterization of the activity of the exogenous 1MeTIQ in the mammalian brain, especially in the nigrostriatal dopaminergic system. On the basis of these studies, the following conclusions may be drawn:

- exogenous 1MeTIQ has high affinity for brain tissue
- 1MeTIQ is a partial dopamine agonist
- 1MeTIQ reversibly inhibits MAOA and MAOB: in vitro and in vivo studies
- 1MeTIQ is a scavenger of free radicals
- 1MeTIQ expresses neuroprotective properties
- 1MeTIQ demonstrates antiaddictive potency

This ability of 1MeTIQ may be of clinical importance and raises hope for its application in neurodegenerative diseases (e.g., Parkinson’s disease) and addiction evoked by drugs of abuse.
